# Chemotherapy After Diagnosis of Malignant Bowel Obstruction is Associated with Superior Survival for Medicare Patients with Advanced Malignancy

**DOI:** 10.1245/s10434-021-09831-0

**Published:** 2021-04-07

**Authors:** Sarah B. Bateni, Alicia A. Gingrich, Amanda R. Kirane, Candice A. M. Sauder, Sepideh Gholami, Richard J. Bold, Frederick J. Meyers, Robert J. Canter

**Affiliations:** 1 Division of Surgical Oncology, Department of Surgery, University of Toronto, Toronto, ON Canada; 2 Division of Surgical Oncology, Department of Surgery, UC Davis Medical Center, University of California, Sacramento, CA USA; 3 Division of Hematology/Oncology, Department of Internal Medicine, UC Davis Medical Center, University of California, Sacramento, CA USA

## Abstract

**Background:**

Although malignant bowel obstruction (MBO) often is a terminal event, systemic therapies are advocated for select patients to extend survival. This study aimed to evaluate factors associated with receipt of chemotherapy after MBO and to determine whether chemotherapy after MBO is associated with survival.

**Methods:**

This retrospective cohort study investigated patients 65 years of age or older with metastatic gastrointestinal, gynecologic, or genitourinary cancers who were hospitalized with MBO from 2008 to 2012 using the Surveillance, Epidemiology, and End Results (SEER)-Medicare database. Fine and Gray models were used to identify factors associated with receipt of chemotherapy accounting for the competing risk of death. Cox models identified factors associated with overall survival.

**Results:**

Of the 2983 MBO patients, 39% (*n* = 1169) were treated with chemotherapy after MBO. No differences in receipt of chemotherapy between the surgical and medical patients were found in the univariable analysis (subdistribution hazard ratio [SHR], 0.96; 95% confidence interval [CI], 0.86–1.07; *p* = 0.47) or multivariable analysis (SHR, 1.12; 95% CI, 1.00–1.26; *p* = 0.06). Older age, African American race, medical comorbidities, non-colorectal and non-ovarian cancer diagnoses, sepsis, ascites, and intensive care unit stays were inversely associated with receipt of chemotherapy after MBO (*p* < 0.05). Chemotherapy with surgery was associated with longer survival than surgery (adjusted hazard ratio [aHR], 2.97; 95% CI, 2.65–3.34; *p* < 0.01) or medical management without chemotherapy (aHR, 4.56; 95% CI, 4.04–5.14; *p* < 0.01). Subgroup analyses of biologically diverse cancers (colorectal, pancreatic, and ovarian) showed similar results, with greater survival related to chemotherapy (*p* < 0.05).

**Conclusions:**

Chemotherapy plays an integral role in maximizing oncologic outcome for select patients with MBO. The data from this study are critical to optimizing multimodality care for these complex patients.

**Supplementary Information:**

The online version contains supplementary material available at 10.1245/s10434-021-09831-0.

Malignant bowel obstruction (MBO) is common among patients with metastatic cancer, occurring in up to 28% of patients with gastrointestinal cancer and 51% of patients with gynecologic cancers.^1^ It often is considered a terminal event because median life expectancy after MBO diagnosis is reported to be shorter than 1 year.^1^–^5^ As such, an increasing focus is on palliative treatment for these patients, with the goals of maximizing symptom relief (i.e., resolution of nausea, vomiting, and abdominal pain) and minimizing therapeutic morbidity in an attempt to optimize patient well-being and quality of life near the end of life.^6^–^9^ However, management strategies are variable depending on institutional practices, physician and/or surgeon preferences, and patients’ goals of care. Treatment of MBO often involves supportive medical care (i.e., gastric decompression and pharmacologic symptom palliation) and procedural interventions (i.e., endoscopic stenting and percutaneous gastrostomy tube placement), with clinicians occasionally advocating for surgical therapies (i.e., enterolysis, resection, bypass, and stoma formation).^7^,^10^–^19^

In light of advancements in systemic therapies for treatment of metastatic disease,^20^–^23^ palliative systemic anti-cancer therapy also may be advocated for select patients with MBO in hopes of prolonging life.^24^ This is especially important in light of research suggesting that a small subset of MBO patients, as large as 20% in some series, experience survival longer than 1 year.^5^,^25^

Considerations of systemic therapy further complicate decision-making for the acute management of MBO. Some physicians may recommend surgery to relieve the immediate obstruction, with the goal of facilitating systemic therapy. However, other data suggest that surgery, with high rates of postoperative serious complications and mortality, may lead to complications that may worsen performance status, impair quality of life, and prevent delivery of systemic therapies.^17^,^25^–^27^

To our knowledge, no research to date has investigated the association between medical and surgical management of MBO and receipt of systemic therapy to assist in this difficult decision-making process. Therefore, this study aimed to conduct a population-based investigation of MBO patients, examining patterns of care after diagnosis of MBO including patient and treatment-related factors associated with the receipt of chemotherapy after diagnosis of MBO and the association of chemotherapy with overall survival (OS).

## Methods

This retrospective cohort study analyzed patients 65 years of age or older with a diagnosis of advanced cancer from 1 January 2008 to 31 December 2012 who were hospitalized with a diagnosis of malignant bowel obstruction (MBO) using Surveillance, Epidemiology, and End Results (SEER)-Medicare-linked datafiles. The research protocol was reviewed and deemed exempt from review by the University of California, Davis Institutional Review Board. The study followed the Strengthening the Reporting of Observational Studies in Epidemiology (STROBE) reporting guidelines.

The study identified 4349 patients 65 years of age or older who met the following criteria: (1) primary gastrointestinal (i.e., colorectal, pancreatic, hepatobiliary, gastric, small intestine, appendiceal), gynecologic (i.e. ovarian, uterine, endometrial), or bladder cancer, (2) stage 4 disease at the time of diagnosis based on American Joint Committee on Cancer (AJCC) sixth- and seventh-edition staging,^28^,^29^ except for ovarian cancer (see later), and (3) hospitalization with a bowel obstruction diagnosis based on the International Classification of Diseases, ninth edition (ICD-9) codes 5608, 56081, 56089, and 5609 within 2 years after the cancer diagnosis.^3^ For ovarian cancer, the study also included patients with T3b and T3c disease, defined as macroscopic peritoneal disease.

The study excluded patients without continuous Medicare parts A and B coverage for 12 months after MBO diagnosis or until death if sooner (*n* = 1225) to ensure complete documentation of medical records and treatment. Those who underwent surgery during their hospitalization for indications unrelated to MBO (*n* = 49) and those transferred to an outside hospital (*n* = 92) also were excluded from analysis.

**Fig. 1:**
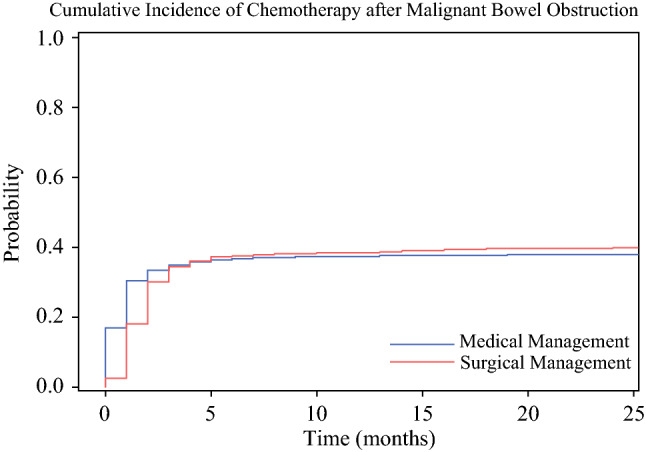
Cumulative incidence of chemotherapy after surgical or medical management of malignant bowel obstruction. Receipt of chemotherapy after malignant bowel obstruction (MBO) diagnosis did not differ between the medical and surgical patients (SHR, 0.96; 95% CI, 0.86–1.07; *p* = 0.47). SHR, subdistribution hazard ratio; CI, confidence interval

The final cohort consisted of 2983 patients (Fig. 1S). Patient demographics and clinicopathologic characteristics extracted from the SEER-Medicare datafiles included age, sex, race, cancer type, presence of ascites and sepsis at admission, and patient comorbidities. The Elixhauser Comorbidity Index was used to assess patient risk associated with comorbidities from ICD-9 diagnoses.^30^–^32^ Chemotherapy and radiotherapy data were obtained from Medicare files using Healthcare Common Prodecure Coding System (HCPCS) and ICD-9 procedure and diagnosis codes.^33^–^35^ Patients were classified as “medical” versus “surgical” management if they did or did not undergo an abdominal operation commonly performed for bowel obstruction during their initial hospitalization for MBO.^25^ The operations included exploratory laparotomy or laparoscopy, open or laparoscopic gastrostomy, gastrectomy, gastric bypass procedure (i.e., gastrojejunostomy), intestinal bypass, small or large bowel resection, and small or large bowel ostomy. As noted earlier, the study excluded 49 patients who had surgery during their hospitalization for indications unrelated to MBO, including spinal and orthopedic procedures.

The primary outcomes for this analysis were receipt of chemotherapy after MBO diagnosis and OS. Chemotherapy after MBO diagnosis included chemotherapy provided during the primary MBO hospitalization and after discharge. Overall survival was calculated from MBO diagnosis to the date of death or the last follow-up visit. The secondary outcomes were complications during the primary MBO hospitalization and within 30-days after discharge, intensive care unit (ICU) stays, 30- and 90-day hospital readmissions, recurrent obstruction, and disposition (including in-hospital death).

### Statistical Analysis

The univariable analysis compared patient demographics, clinicopathologic characteristics, and treatment outcomes using chi-square tests for categorical variables, Student’s *t* tests for normally distributed continuous variables, and Mann-Whitney *U* tests for non-normally distributed continuous variables. Uni- and multivariable Fine and Gray competing-risk models were used to compare the cumulative incidence of chemotherapy between the surgical and medical patients after MBO diagnosis accounting for competing risks of death.^36^,^37^ Data for competing-risk models are presented as subdistribution hazard ratios (SHRs) and adjusted SHRs (aSHRs).

The covariates in the initial multivariable competing-risk model were age, race, sex, Elixhauser Comorbidity Index, cancer diagnosis, sepsis and/or ascites, re-obstruction, complications, and intensive care unit (ICU) stay. A second competing-risk model was performed with complications, ICU stays, and re-obstruction removed because these outcomes were associated with the primary management of the MBO (i.e., surgical or medical), and we were concerned that the initial model was potentially over-controlled with respect to surgical and medical management.

Cox proportional hazards uni- and multivariable analyses were performed to compare OS among surgical and medical patients who did and did not undergo chemotherapy.^38^ The covariates in the multivariable survival models were age, race, sex, Elixhauser Comorbidity Index, cancer diagnosis, sepsis and/or ascites, and radiation therapy. Subgroup survival analysis was performed for the three most common cancer diagnoses (colorectal [*n* = 1459], pancreatic [*n* = 491], and ovarian [*n* = 406] cancer) given their different natural histories and differential responses to systemic therapy. Statistical analyses were performed using SAS 9.4 (SAS Institute Inc, Cary, NC, USA). All tests were two-sided, and *p* values lower than 0.05 were considered statistically significant.

## Results

Of the 2983 patients hospitalized for MBO from 2008 to 2012, 1511 underwent surgical management and 1472 underwent medical management. As shown in Table 1, the two groups had significant demographic and clinicopathologic differences. Those who underwent surgery were slightly older (77.8 vs 76.9 years; *p* < 0.01), more frequently were male (42.5% vs 38.4%; *p* = 0.02), had higher Elixhauser Comorbidity Index scores (20.6 vs 19.5; *p* < 0.01), and less frequently presented with ascites (19.7% vs 24.3%; *p* < 0.01) or sepsis (2.2% vs 4.3%; *p* < 0.01). The surgical patients more commonly had colorectal cancer (60.6% vs 36.9%) and less frequently had pancreatic (11.1% vs 22.0%), ovarian (10.7% vs 16.6%), or gastric (2.3% vs 7.3%; *p* < 0.01) cancers. The patients who underwent medical management had a longer time from cancer diagnosis to MBO diagnosis (median, 3 months; interquartile range [IQR], 0–9 months vs 0 months; IQR, 0–1 months; *p* < 0.01).

**Table 1:** Patient demographics and clinicopathologic characteristics

	Surgical management	Medical management	*p* value
	(*n* = 1511)	(*n* = 1472)	
	*n* (%)	*n* (%)	
Mean age (years)	77.8 ± 7.4	76.9 ± 7.2	< 0.01
Male sex	642 (42.5)	565 (38.4)	0.02
*Race*			
White	1269 (84.0)	1201 (81.6)	0.12
African American	151 (10.0)	153 (10.4)	
Asian/Pacific Islander	83 (5.5)	113 (7.7)	
Other	<11 (<0.7)	< 11 (<0.7)	
Mean Elixhauser Comorbidity Index	20.6 ± 8.8	19.5 ± 9.7	< 0.01
Ascites^a^	297 (19.7)	357 (24.3)	< 0.01
Sepsis^a^	33 (2.2)	63 (4.3)	< 0.01
*Primary cancer diagnosis*			< 0. 01
Colorectal	916 (60.6)	543 (36.9)	
Pancreatic	167 (11.1)	324 (22.0)	
Ovarian	162 (10.7)	244 (16.6)	
Small intestine	118 (7.8)	53 (3.6)	
Gastric	35 (2.3)	107 (7.3)	
Uterine	27 (1.8)	60 (4.1)	
Biliary	21 (1.4)	61 (4.1)	
Hepatic	<11 (<0.7)	26 (1.8)	
Appendiceal	30 (2.0)	31 (2.1)	
Bladder	29 (1.9)	23 (1.6)	
Median months from cancer diagnosis to MBO (IQR)	0 (0–1)	3 (0–9)	< 0.01
Chemotherapy before MBO	267 (17.7)	723 (49.1)	< 0.01
Chemotherapy after MBO	608 (40.2)	561 (38.1)	0.23
NCI cancer center	66 (4.4)	124 (8.4)	< 0. 01
Teaching hospital	758 (50.2)	784 (53.1)	0.14

*MBO* malignant bowel obstruction, *IQR* interquartile range, *NCI* National Cancer Institute

^a^Present at admission

Among the patients who underwent surgery, the most frequent operation performed was bowel resection (*n* = 778, 51.5%), followed by ostomy (*n* = 625, 41.4%) and entero-entero bypass (*n* = 275, 18.2%). Most of the patients were treated at a cancer center not designated by the National Cancer Institute (NCI), with only 8.4% of the medical and 4.4% of the surgical patients treated at an NCI cancer center (*p* < 0.01).

Compared with the medical patients, the surgical patients had higher rates of complications during their index hospitalization and within 30 days after discharge (23.4% vs 17.7%; *p* < 0.01), more ICU stays (50.0% vs 17.5%; *p* < 0.01), and longer hospital stays (11 vs 5 days; *p* < 0.01) (Table S1). The surgical patients also had higher rates of disposition to nursing/rehab facilities (27.0% vs 11.3%), lower rates of disposition to home (48.2% vs 53.5%), and lower rates of disposition to hospice (11.9% vs 20.9%) than the medical patients (*p* < 0.01). In contrast, the medical patients had higher rates of 30-day readmission (29.4% vs 22.6%; *p* < 0.01) and re-obstruction (23.8% vs 14.8%; *p* < 0.01).

Notably, the rates of chemotherapy after MBO diagnosis did not differ significantly between the surgical and medical patients (40.2% vs 38.1%; *p* = 0.23; Table 1). Additionally, in the Fine and Gray competing-risk models (Table 2), the cumulative incidence of chemotherapy after MBO diagnosis did not differ between the medical and surgical patients in either the univariable (SHR, 0.96; 95% CI, 0.86–1.07; *p* = 0.47; Fig. 1) or multivariable (aSHR, 1.12; 95% CI, 1.00–1.26; *p* = 0.06) analysis.

**Table 2:** Fine and Gray competing-risks uni- and multivariable models of demographic and clinical characteristics associated with chemotherapy after malignant bowel obstruction diagnosis

	Univariable analysis			Multivariable analysis		
	SHR	95% CI	*p* value	Adjusted SHR	95% CI	*p* value
Age	0.94	0.93–0.95	< 0.01	0.94	0.94–0.95	< 0.01
*Sex*						
Male	Reference			Reference		
Female	0.98	0.88–1.10	0.77	0.93	0.83–1.05	0.24
*Race*						
White	Reference			Reference		
African American	0.76	0.62–0.92	< 0.01	0.72	0.59–0.87	< 0.01
Asian/Pacific Islander	1.03	0.82–1.28	0.82	1.00	0.81–1.23	0.99
Other	0.71	0.29–1.78	0.47	0.56	0.21–1.50	0.25
Elixhauser Comorbidity Index	0.98	0.97–0.98	< 0.01	0.99	0.98–0.99	< 0.01
*Primary cancer diagnosis*						
Colorectal	Reference			Reference		
Ovarian	1.48	1.28–1.71	< 0.01	1.49	1.27–1.75	< 0.01
Pancreatic	0.80	0.67–0.95	0.01	0.83	0.70–0.99	0.03
Small intestine	0.93	0.73–1.17	0.54	0.76	0.61–0.95	0.02
Gastric	0.72	0.53–0.99	0.04	0.65	0.47–0.90	0.01
Uterine	1.06	0.76–1.47	0.73	0.99	0.70–1.39	0.94
Biliary	0.79	0.53–1.19	0.26	0.73	0.49–1.09	0.13
Hepatic	0.69	0.36–1.30	0.25	0.73	0.37–1.44	0.37
Bladder	0.50	0.28–0.90	0.02	0.49	0.28–0.87	0.02
Appendiceal	1.43	1.06–1.94	0.02	1.01	0.75–1.37	0.91
Sepsis	0.30	0.17–0.51	< 0.01	0.37	0.22–0.64	< 0.01
Ascites	0.78	0.67–0.90	< 0.01	0.74	0.64–0.85	< 0.01
ICU stay	0.65	0.58–0.73	< 0.01	0.74	0.65–0.84	< 0.01
Complications	0.92	0.82–1.06	0.25	0.88	0.77–1.00	0.06
Re-obstruction	2.41	2.15–2.70	< 0.01	1.97	1.75–2.22	< 0.01
*Management*						
Medical	Reference			Reference		
Surgery	0.96	0.86–1.07	0.47	1.12	1.00–1.26	0.06

*SHR* subdistribution hazard ratio, *CI* confidence interval, *ICU* intensive care unit

In the second competing-risk model, with complications, ICU stays, and re-obstruction removed, there continued to be no difference in chemotherapy after MBO (aSHR, 0.96; 95% CI, 0.86–1.07; *p* = 0.44). Older age, African American race, higher Elixhauser Comorbidity Score, presence of sepsis and ascites at admission, and ICU stays all were inversely associated with receipt of chemotherapy after MBO in both the uni- and multivariable analyses (Table 2, *p* < 0.05 all).

The rates of chemotherapy varied by cancer type, with chemotherapy administered after MBO diagnosis to 54.1% of those with appendiceal cancer, 52% of those with ovarian cancer, 40.2% of those with uterine cancer, 40% of those with colorectal cancer, 38% of those with small intestine cancer, 31.6% of those with pancreatic cancer, 30.5% of those with biliary cancer, 28.9% of those with gastric cancer, 28.1% of those with hepatic cancer, and 21.2% of those with bladder cancer (*p* < 0.01).

**Fig. 2:**
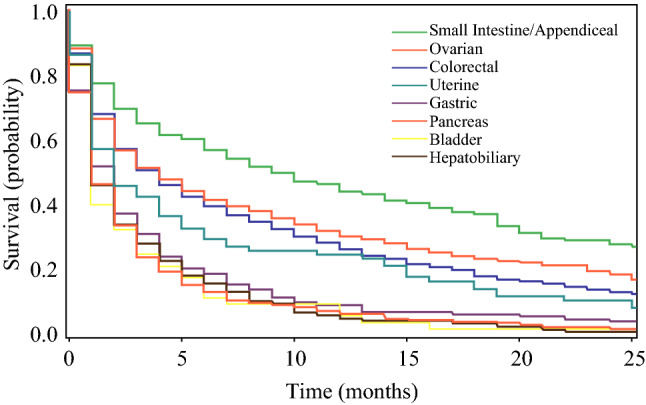
Survival by cancer diagnosis

As shown in Fig. 2, survival differed significantly by cancer type. The patients with primary appendiceal or small intestinal cancers had the longest median survival (9.5 months; IQR, 2–30 months), followed by those with ovarian cancer (4 months; IQR 1–17 months) and colorectal cancer (3 months; IQR, 1–13 months), then by those with gastric cancer (2 months; IQR, 1–4 months) and uterine cancer (2 months; IQR, 1–11 months), and finally by those with hepatobiliary cancer (1 month; IQR, 1–4 months), bladder cancer (1 month; IQR, 1–3.5 months), and pancreatic cancer (1 month; IQR, 0–3 months). Overall, 22.9% of the patients were alive at 1 year.

**Fig. 3:**
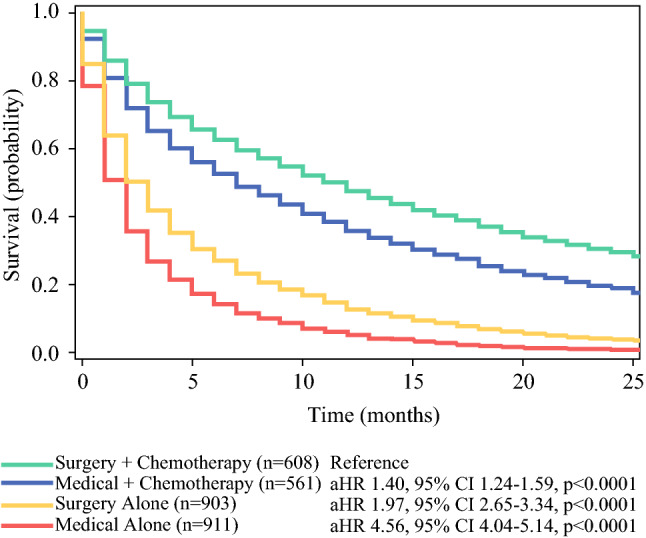
Overall survival by treatment of malignant bowel obstruction adjusted for patient demographic and clinicopathologic characteristics. aHR, adjusted hazard ratio; CI confidence interval

Both the uni- and multivariable analyses (Fig. 3; Table S1), showed worse survival for the patients who had medical management with chemotherapy (HR, 1.43; 95% CI, 1.27–1.61; *p* < 0.01; aHR, 1.40; 95% CI, 1.24–1.59; *p* < 0.01), surgery alone (HR, 2.94; 95% CI, 2.63–3.28; *p* < 0.01; aHR, 2.97; 95% CI, 2.65–3.34; *p* < 0.01), and medical management alone (HR, 4.91; 95% CI, 4.38–5.49; *p* < 0.01; aHR, 4.56; 95% CI, 4.04–5.14; *p* < 0.01) than for those who had surgery with chemotherapy. Longer survival was associated with female sex (aHR, 0.90; 95% CI, 0.83–0.98; *p* = 0.01) and radiotherapy (aHR, 0.85; 95% CI, 0.76–0.96; *p* < 0.01), whereas worse survival was associated with higher Elixhauser Comorbidity Score (aHR, 1.01; 95% CI, 1.01–1.02; *p* < 0.01) and ascites (aHR, 1.36; 95% CI, 1.24–1.49; *p* < 0.01).

**Fig. 4:**
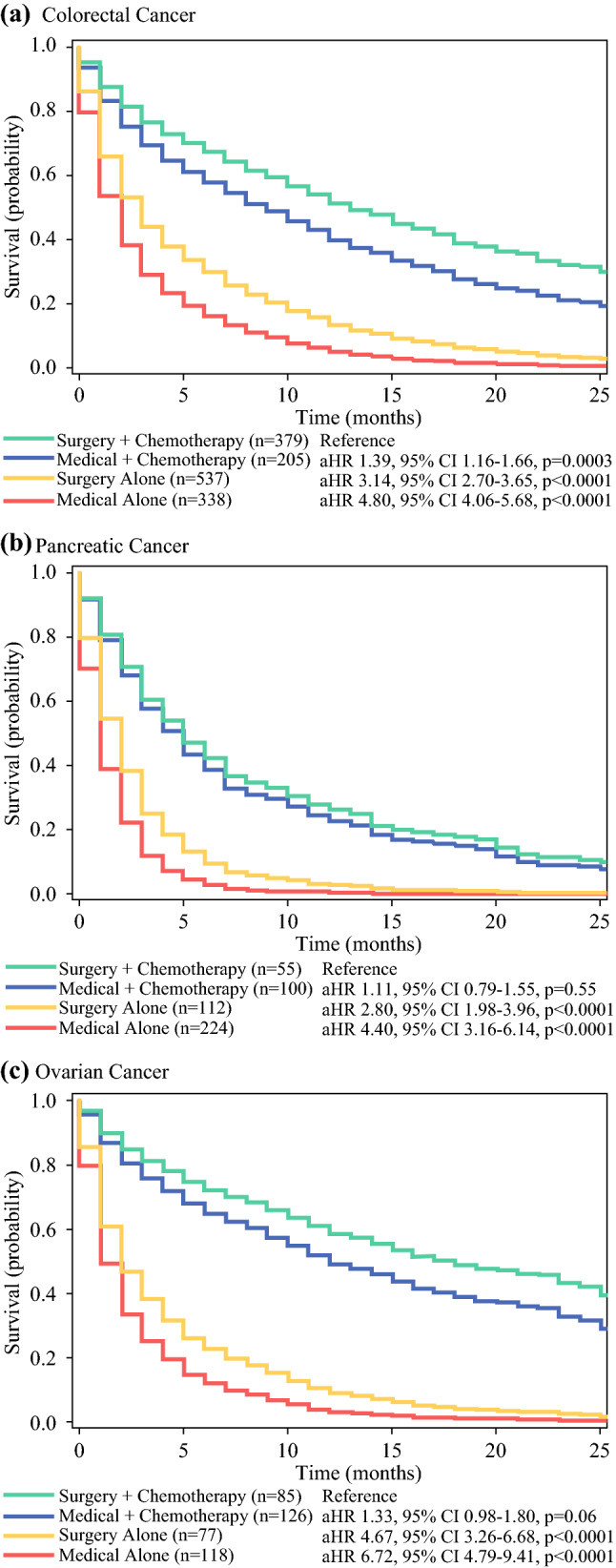
Subgroup analysis of overall survival by treatment of malignant bowel obstruction adjusted for patient demographics and clinicopathologic characteristics for (**a**) colorectal, (**b**) pancreatic, and (**c**) ovarian cancers

Similar results were observed in a subgroup analysis by cancer type (i.e., colorectal, pancreatic, and ovarian cancer; Fig. 4). For colorectal cancer, medical management with chemotherapy (aHR, 1.39; 95% CI, 1.16–1.66; *p* < 0.01), surgery alone (aHR, 3.14; 95% CI, 2.70–3.65; *p* < 0.01), and medical management alone (aHR, 4.80; 95% CI, 4.06–5.68; *p* < 0.01) were associated with worse survival than surgery with chemotherapy. However, for pancreatic and ovarian cancers, surgery and medical management with chemotherapy were associated with longer survival than medical or surgical management alone (*p* < 0.05; Fig. 4; Table S2). Survival did not differ between surgery with chemotherapy and medical management with chemotherapy for either pancreatic cancer (aHR, 1.11; 95% CI, 0.79–1.55; *p* = 0.55) or ovarian cancer (aHR, 1.33; 95% CI, 0.98–1.80; *p* = 0.07; Fig. 4).

## Discussion

The current study identified important clinical and treatment-related factors associated with receipt of chemotherapy and survival after MBO diagnosis. Most notably and unexpectedly, we observed that chemotherapy after diagnosis and treatment of index MBO was associated with clinically and statistically significant longer survival regardless of tumor type and independent of how the index MBO was treated for select tumor types. Undoubtedly, selection bias played a significant role in these results as did tumor biology, but the consistently favorable association of chemotherapy with improved outcomes for both typically chemo-resistant cancers (e.g., pancreas and bladder cancer) and more chemo-sensitive cancers (e.g., colorectal cancer) suggests that a more aggressive approach to cancer care for these patients may translate to a survival benefit. In fact, we observed a 22.9% 1-year survival for our cohort overall, which is notable given that this was an elderly population biased to less favorable outcomes than other patient populations.

Although recent analyses have emphasized a more conservative approach for patients with MBO focused on de-escalation of care to avoid therapeutic morbidity,^25^,^39^ our data also suggest that attention should be paid to avoiding potential undertreatment of these patients, recognizing that chemotherapy may be critical to optimal cancer-related outcomes. The decision to provide more aggressive care for terminal cancer patients remains complex,^40^,^41^ and careful patient selection plays a key role, as our results suggest that younger, healthier, and colorectal cancer patients are more likely to receive chemotherapy after MBO, all of which may have biased the results.

Although systemic therapies are the backbone of treatment for patients with metastatic cancer,^20^–^23^,^42^–^44^ the data applying these therapies to patients after an MBO diagnosis are limited. Chouhan et al.^45^ performed a retrospective analysis of 82 patients with MBO and carcinomatosis treated with total parental nutrition and chemotherapy at a single institution. They noted that only 23% of the patients responded to chemotherapy, but that the response to chemotherapy was associated with greater survival. The findings from the current study suggest similarly that select patients may benefit from systemic therapy, with a clinically meaningful survival benefit independent of how the index MBO was treated. However, the decision to initiate systemic therapy must not be taken lightly. Pertinent factors regarding prognosis and end-of-life wishes must also be taken into consideration because not all patients may respond to chemotherapy or wish to pursue aggressive care toward the end of life as such therapies can have an impact on quality of life.^41^,^45^–^47^

To guide clinicians with this process, we identified important patient characteristics associated with both receipt of chemotherapy and survival after MBO including fewer medical comorbidities, absence of ascites, and a colorectal cancer diagnosis. To our knowledge, this is the first population-based study to examine clinical and treatment-related factors associated with the initiation of chemotherapy after MBO. Helyer et al.^24^ performed a single-institution analysis of colorectal patients with MBO who underwent surgical intervention and subsequent palliative chemotherapy therapy. They observed that 16 (34%) of the 47 patients in their study received palliative chemotherapy after surgery. Because longer survival also was associated with fewer medical comorbidities, a colorectal cancer diagnosis, and absence of ascites, oncologists should incorporate these factors into their decision-making when recommending chemotherapy in MBO patients.

It also is important to note that although chemotherapy was consistently associated with longer survival among MBO patients, the role of surgery remains unclear. This was evident in our subgroup analysis, which found that chemotherapy with or without surgery was associated with improved survival for pancreatic and ovarian cancers, whereas chemotherapy with surgery was associated with longer survival than all other treatments for colorectal cancer. These findings suggest that although surgery may play a role for MBO patients with colorectal cancer, it may not play a role for other cancers. These findings are consistent with prior research showing equivalent overall survival for MBO patients treated with surgery and those treated with supportive medical care.^4^,^25^,^39^,^48^,^49^ Such results highlight the impact of tumor biology on outcomes for these challenging and heterogeneous patients and also reinforces the pitfalls of a one-size-fits-all approach. Therefore, the decision to initiate chemotherapy should not be dependent on the patient also undergoing surgery because these decisions should be considered independent of one another. This approach is further supported by the finding that surgery was not associated with a greater likelihood for the receipt of subsequent chemotherapy.

Overall, our findings further underscore the complexity of decision-making for MBO patients because survival outcomes varied markedly, with a median survival of 2 to 4 months, but with 22.9% of this elderly cohort living longer than 1 year. As such, a multidisciplinary team with expertise in advanced cancer is crucial to ensure that the course of treatment weighs the patient’s clinical factors, tumor biology, prognosis, and goals of care, with the goal of maximizing oncologic outcomes while minimizing treatment-related morbidity and mortality. The initiation of multidisciplinary team approaches for MBO patients has been associated with improved outcomes including shorter cumulative hospital stays, less surgical intervention, and longer survival.^50^ Our data also suggest that greater use of palliative chemotherapy after a diagnosis and management of MBO may contribute to more favorable survival outcomes.

It is important to acknowledge the limitations of this study. By using SEER-Medicare, we lacked some important patient details including patient functional status as well as laboratory and imaging findings. Although we attempted to use surrogate markers for patient functional status and laboratory findings, unmeasured confounders likely remained in our analyses. In addition, we lacked knowledge about patients’ end-of-life goals of care and wishes, and these are paramount in the clinical decision-making for complex patients such as these. Similarly, we were unable to assess patients’ quality of life among the various subgroups, which is a critical consideration for advanced cancer patients, including those with MBO. Finally, although we were able to identify a large population of patients with MBO, all the patients were 65 years of age or older, so these findings may not be applicable to younger cohorts. Despite these limitations, our findings remain clinically relevant and unexpected because we were able to identify a large cohort of U.S. patients with MBO who demonstrated a clinically meaningful association between receipt of chemotherapy and survival after MBO despite labeling of MBO as a potentially “terminal event.”

In conclusion, despite the tendency for a poor prognosis after a diagnosis of MBO in advanced malignancy, chemotherapy after MBO is associated with a superior oncologic outcome for a significant subset of patients. Careful patient selection remains essential in the identification of patients who may benefit from “aggressive care” toward the end of life. Because decision-making for this vulnerable population continues to be complex, multidisciplinary care remains fundamental to ensure that multimodality therapy aligns closely with the patient’s clinical status and goals of care.

## Supplementary Information

Below is the link to the electronic supplementary material.

Supplementary file 1 (DOCX 143 kb)
