# Supplementary material for: Chemotherapy After Diagnosis of Malignant Bowel Obstruction is Associated with Superior Survival for Medicare Patients with Advanced Malignancy
Source: Ann Surg Oncol. 2021 Apr 7;28(12):7555–63. doi: 10.1245/s10434-021-09831-0 (PMC8519893; doi:10.1245/s10434-021-09831-0)
Supplement: Supplementary file 1 — (DOCX 143 kb) [file 10434_2021_9831_MOESM1_ESM.docx]

Supplement

**Figure 1S.** Consort diagram.



**Table 1S.** Univariable Outcomes for Surgical compared to Medical Management of Malignant Bowel Obstruction

|  | Surgical Management | Medical Management |  |
| --- | --- | --- | --- |
|  | N=1511 | N=1472 |  |
|  | N (%) or Median (IQR) | N (%) or Median (IQR) | P value |
| Complications (N, %)* | 354 (23.4%) | 261 (17.7%) | p<0. 01 |
| Hospital length of stay, days (median, IQR) | 11 (8-11) | 5 (3-9) | p<0. 01 |
| ICU stay (N, %) | 755 (50.0%) | 258 (17.5%) | p<0. 01 |
| Disposition (N, %) |  |  | p<0. 01 |
| *Home* | 729 (48.2%) | 788 (53.5%) |  |
| *Hospice* | 180 (11.9%) | 307 (20.9%) |  |
| *Nursing/Rehab Facility* | 408 (27.0%) | 167 (11.3%) |  |
| *Other* | 49 (3.2%) | 27 (1.8%) |  |
| *In-hospital death* | 145 (9.6%) | 183 (12.4%) |  |
| Readmission 30-day** (N, %) | 309 (22.6%) | 379 (29.4%) | p<0. 01 |
| Readmission 90-day** (N, %) | 509 (37.3%) | 515 (40.0%) | p=0.15 |
| Reobstruction** (N, %) | 202 (14.8%) | 307 (23.8%) | p<0. 01 |
| Chemotherapy after MBO diagnosis (N, %) | 608 (40.2%) | 561 (38.1%) | p=0.23 |
| Months from MBO diagnosis to chemotherapy*** (median, IQR) | 1 (0-1) | 2 (1-3) | p<0.01 |
| Radiotherapy after MBO diagnosis (N, %) | 127 (8.4%) | 78 (5.3%) | p<0.01 |
| Overall survival, months (median, IQR) | 4 (1-16) | 2 (1-6) | p<0. 01 |
| 1-year survival (N, %) | 454 (30.1%) | 228 (15.5%) | p<0.01 |
| SD, standard deviation; MBO, malignant bowel obstruction; IQR, interquartile range; *complications are defined as those during the hospital admission and within 30 days of discharge; **excluding in-hospital death (n=2,655); ***of those who underwent chemotherapy (n=1,169) | | | |

**Table 2S.** Univariable and Multivariable Analysis for Overall Survival among Patients with Malignant Bowel Obstruction.

|  | Univariable Analysis | | | Multivariable Analysis | | |
| --- | --- | --- | --- | --- | --- | --- |
|  | Hazard Ratio | 95% CI | P Value | Adjusted Hazard  Ratio | 95% CI | P Value |
| Age | 1.02 | 1.02-1.03 | <0.01 | 1.00 | 1.00-1.01 | 0.26 |
| Sex |  |  |  |  |  |  |
| *Male* | Reference |  |  | Reference |  |  |
| *Female* | 0.95 | 0.88-1.02 | 0.18 | 0.90 | 0.83-0.98 | 0.01 |
| Race |  |  |  |  |  |  |
| *White* | Reference |  |  | Reference |  |  |
| *African American* | 1.10 | 0.97-1.24 | 0.12 | 1.00 | 0.88-1.13 | 1.00 |
| *Asian/Pacific Islander* | 1.13 | 0.98-1.31 | 0.11 | 1.09 | 0.94-1.26 | 0.27 |
| *Other* | 0.75 | 0.42-1.36 | 0.34 | 0.73 | 0.40-1.32 | 0.29 |
| Elixhauser Comorbidity Index | 1.02 | 1.02-1.02 | <0.01 | 1.01 | 1.01-1.02 | <0.01 |
| Primary Cancer Diagnosis |  |  |  |  |  |  |
| *Colorectal* | Reference |  |  | Reference |  |  |
| *Ovarian* | 0.90 | 0.81-1.01 | 0.08 | 0.94 | 0.83-1.06 | 0.32 |
| *Pancreatic* | 1.88 | 1.69-2.09 | <0.01 | 1.62 | 1.46-1.81 | <0.01 |
| *Small Intestine* | 0.64 | 0.54-0.76 | <0.01 | 0.54 | 0.45-0.64 | <0.01 |
| *Gastric* | 1.61 | 1.35-1.92 | <0.01 | 1.19 | 0.99-1.42 | 0.06 |
| *Uterine* | 1.23 | 0.99-1.53 | 0.07 | 1.23 | 0.98-1.54 | 0.08 |
| *Biliary* | 1.81 | 1.45-2.27 | <0.01 | 1.43 | 1.14-1.79 | <0.01 |
| *Hepatic* | 1.79 | 1.26-2.54 | <0.01 | 1.29 | 0.91-1.85 | 0.16 |
| *Bladder* | 1.85 | 1.40-2.44 | <0.01 | 1.54 | 1.16-2.04 | <0.01 |
| *Appendiceal* | 0.74 | 0.57-0.96 | 0.02 | 0.69 | 0.53-0.89 | <0.01 |
| Sepsis | 1.56 | 1.26-1.91 | <0.01 | 1.06 | 0.86-1.31 | 0.60 |
| Ascites | 1.45 | 1.32-1.58 | <0.01 | 1.36 | 1.24-1.49 | <0.01 |
| Radiotherapy | 0.72 | 0.64-0.81 | <0.01 | 0.85 | 0.76-0.96 | <0.01 |
| Management |  |  |  |  |  |  |
| *Surgery + Chemotherapy* | Reference |  |  | Reference |  |  |
| *Medical + Chemotherapy* | 1.43 | 1.27-1.61 | <0.01 | 1.40 | 1.24-1.59 | <0.01 |
| *Surgery Alone* | 2.94 | 2.63-3.28 | <0.01 | 2.97 | 2.65-3.34 | <0.01 |
| *Medical Alone* | 4.91 | 4.38-5.49 | <0.01 | 4.56 | 4.04-5.14 | <0.01 |
| CI, confidence interval; | | | | | | |

**Table 3S.** Multivariable Cox Proportional Hazards Pairwise Comparisons of Overall Survival for Pancreatic and Ovarian Cancer Subgroup Analysis

| Management | Pancreatic Cancer | | Ovarian Cancer | |
| --- | --- | --- | --- | --- |
|  | aHR | 95% CI | aHR | 95% CI |
| *Medical + Chemotherapy vs. Surgery + Chemotherapy* | 1.11 | 0.79-1.55 | 1.33 | 0.98-1.80 |
| *Surgery Alone vs. Surgery + Chemotherapy* | 2.80* | 1.98-3.98 | 4.67* | 3.26-6.68 |
| *Medical Alone vs. Surgery + Chemotherapy* | 4.40* | 3.16-6.14 | 6.72* | 4.79-9.41 |
| *Surgery Alone vs. Medical + Chemotherapy* | 2.53* | 1.86-3.44 | 3.51* | 2.49-4.95 |
| *Medical Alone vs. Medical + Chemotherapy* | 3.97* | 2.99-5.29 | 5.05* | 3.69-6.90 |
| *Medical Alone vs. Surgery Alone* | 1.57* | 1.24-2.00 | 1.44* | 1.07-1.93 |

aHR, adjusted hazard ratio; CI, confidence interval; Model covariates included age, Elixhauser comorbidity index, sex, face, ascites, sepsis on presentation, and radiation therapy. *statistically significant with p<0.05.
